# Difference Between Signet Ring Cell Gastric Cancers and Non-Signet Ring Cell Gastric Cancers: A Systematic Review and Meta-Analysis

**DOI:** 10.3389/fonc.2021.618477

**Published:** 2021-05-07

**Authors:** Chi Zhang, Ran Liu, Wei-Han Zhang, Xin-Zu Chen, Kai Liu, Kun Yang, Xiao-Long Chen, Lin-Yong Zhao, Zhi-Xin Chen, Zong-Guang Zhou, Jian-Kun Hu

**Affiliations:** ^1^Department of Gastrointestinal Surgery, Laboratory of Gastric Cancer, State Key Laboratory of Biotherapy, Collaborative Innovation Center for Biotherapy, West China Hospital, Sichuan University, Chengdu, China; ^2^Engineering Research Center of Medical Information Technology, Ministry of Education, West China Hospital, Sichuan University, Chengdu, China; ^3^Department of Gastrointestinal Surgery, Laboratory of Digestive Surgery, State Key Laboratory of Biotherapy, Collaborative Innovation Center for Biotherapy, West China Hospital, Sichuan University, Chengdu, China

**Keywords:** prognosis, stage, sex, gastric cancer, signet ring cell

## Abstract

**Background:** There is controversy about the characteristics and prognostic implications of signet ring cell gastric cancers and non-signet ring cell gastric cancers.

**Objective:** This study aims to evaluate clinicopathological characteristics and prognoses of signet ring cell carcinoma (SRCC) and non-signet ring cell carcinoma (NSRCC) of stomach.

**Methods:** Studies compared between SRCC and NSRCC of the stomach after gastrectomy and published before September 1st, 2020, in the PubMed, Cochrane, and Embase databases, were identified systematically.

**Results:** A total of 2,865 studies were screened, and 36 studies were included, with 19,174 patients in the SRCC group and 55,942 patients in the NSRCC group. SRCC patients were younger in age (*P* < 0.001), less likely to be male patients (*P* < 0.001), more afflicted with upper third lesions (*P* < 0.001), and presenting with more Borrmann type IV tumors (*P* = 0.005) than NSRCC patients. Lymph nodes metastasis was similar between SRCC and NSRCC patients with advanced tumor stage (OR: 0.86, 95% CI: 0.671.10, *P* = 0.23), but lower in the SRCC than NSRCC patients with early tumor stage (OR: 0.73; 95% CI: 0.560.98, *P* = 0.02). SRCC patients had comparable survival outcomes with NSRCC patients for early gastric cancers (HR: 1.05, 95% CI: 0.651.68, *P* < 0.001) but had significantly poor prognosis for patients with advanced tumor stage (HR: 1.50, 95% CI: 1.281.76, *P* < 0.001).

**Conclusions:** Signet ring cell carcinomas of the stomach are an increasingly common histopathological subtype of gastric cancers. These kinds of patients tend to be younger in age and more often female. Although, signet ring cell gastric cancer is a negative prognostic factor for patients with advanced stage. The difference is that for early stage of signet ring cell gastric cancers, it has low lymph nodes metastasis rate and comparable prognosis with non-signet ring cell cancers.

## Introduction

As one of the most common malignancies of the world, gastric cancer has a higher incidence in East Asian countries (1,4). The signet ring cell carcinoma (SRCC) of the stomach is one of histological subtypes of gastric adenocarcinomas. According to the World Health Organization (WHO) histological classification, the SRCC is an adenocarcinoma in which more than 50% of the tumor cells are scattered malignant cells containing intracytoplasmic mucin (5, 6). Besides, in the other histological classification of gastric cancers, SRCC is also can be classified as diffused type by Lauren classification and undifferentiated type by Japanese Gastric Cancer Classification (7, 8). Some studies reported that the SRCC of the stomach has unique and distinct clinicopathological characteristics with other types of carcinomas of the stomach (9,11). Some scholars have stated that the SRCC patients are further younger and include more female patients, while easily have lymph nodes metastasis and distal metastasis than non-signet ring cell carcinoma (NSRCC) patients (11,13). Besides, the prognostic implication of SRCC is still with controversies. Some studies reported that SRCC has better survival outcomes than NSRCC patients (14,16). Also, some studies have presented that the survival outcomes of SRCC were similar and even poorer than NSRCC patients (17,19). With respect to these controversies, some scholars attribute the differences to the different components of the tumor stage between SRCC and NSRCC patients (20).

In view of the foregoing, we performed this study aiming to systematically ascertain and comprehensively clarify the characteristics of signet ring cell gastric cancers. The primary outcomes of this study were the survival outcomes of SRCC patients. Other clinical characteristics, such as age, sex, and tumor stage, were also analyzed.

## Methods and Materials

### Search Strategy and Study selection

A comprehensive literature search was performed in the Web of Knowledge, PubMed/Medline, Cochrane Collaborative Central Register of Controlled Trials, and Embase databases on September 1st, 2020, using the terms gastric cancer, gastric carcinoma, gastric neoplasm, signet ring cell, and restricted to title, abstract, and keywords. Previously published meta-analysis and systematic reviews were searched as well. Relevant articles were manually checked from the reference lists of the retrieved articles. Titles, abstracts, and subsequently full-text articles were screened by two authors (C Zhang and R Liu) based on the inclusion and exclusion criteria of this study.

### Inclusion and Exclusion Criteria

The present study included those studies comparing SRCC with NSRCC (either well-controlled, moderated, and or/poorly differentiated cancers) on at least one outcome of interest. Exclusion criteria included the following: (1) cancers compared only with mucinous carcinoma patients; (2) patients without gastrectomy; (3) patients with endoscopic mucosal resection (EMR) and endoscopic submucosal dissection (ESD); (4) review articles or case reports; (5) articles in other languages than English; and (6) incomplete or duplicate data.

### Data Extraction

The data were independently extracted by two authors (C Zhang and R Liu) from the studies included. For each study, we recorded the name of first author, year of publication, country, study design, the time period of the included patients, classification of SRCC, sample size of SRCC and NSRCC and the definition of NSRCC. The following clinicopathological characteristics were also extracted: age, sex, tumor location, tumor size (cm), differentiated degree of NSRCC group, Borrmann type, invasive depth of tumor (T stage), status of lymph nodes metastasis (N stage), distal metastasis (M stage), TNM stage and postoperative 5-year overall survival. For those studies with more than one article and with duplicated data, only the article having the most complete data was included for analysis.

### Quality Assessment

The quality of studies included was independently assessed by two authors (C Zhang and R Liu), according to the Newcastle-Ottawa Scale (NOS) (21). If there existed disagreement on the assessment, the consensus was reached by a discussion with supervisors (WH Zhang and JK Hu). All of those studies included were ranked with a maximum of 9 points, studies with a NewcastleOttawa Scale score lower than 6 were considered as a moderate or low-quality study.

### Statistical Analysis

The meta-analysis was performed according to the Cochrane guidelines (22). Category data were analyzed using the Mantel-Haenszel method. Continuous data were presented as the mean standard deviation (SD) and analyzed by the inverse variance method. For those studies which only reported median values and ranges for continuous variables, the means and standard deviations were converted according to the method reported by Hozo et al. (23). The odds ratio (OR), mean difference (MD), and hazard ratio (HR) were used to evaluated dichotomous data, continuous data, and survival outcomes, respectively. All of the OR, HR, and MD were reported with 95% confidence intervals (CIs).

Egger's regression and the funnel plot were used to test the publication bias. Heterogeneity was assessed using by the *I*^2^ statistic. When *I*^2^ <30%, it was considered to be low heterogeneity; 30 and <50% were considered to be moderate heterogeneity, and 50% was considered to be considerable heterogeneity. In the case of considerable heterogeneity, the random-effects model was used. For data with low or moderate heterogeneity, the fixed-effects model was used. Subgroup analyses based on different tumor stages were performed to identify potential differences between SRCC and NSRCC patients. The source of heterogeneity was explored with the meta-regression analysis. Possible parameters (publication year, sample size, study region, and tumor stage) were tested to explore potential origin of heterogeneity. All of the statistical analysis was performed by the metafor and meta packages of R software, version 3.2.4 (R Foundation for Statistical Computing, Vienna, Austria) and Review Manager software, version 5.3 (Cochrane, London, UK). A *P*-value <0.05 was considered statistically significant in the present study.

## Results

### Characteristics of the Studies

**Figure 1:**
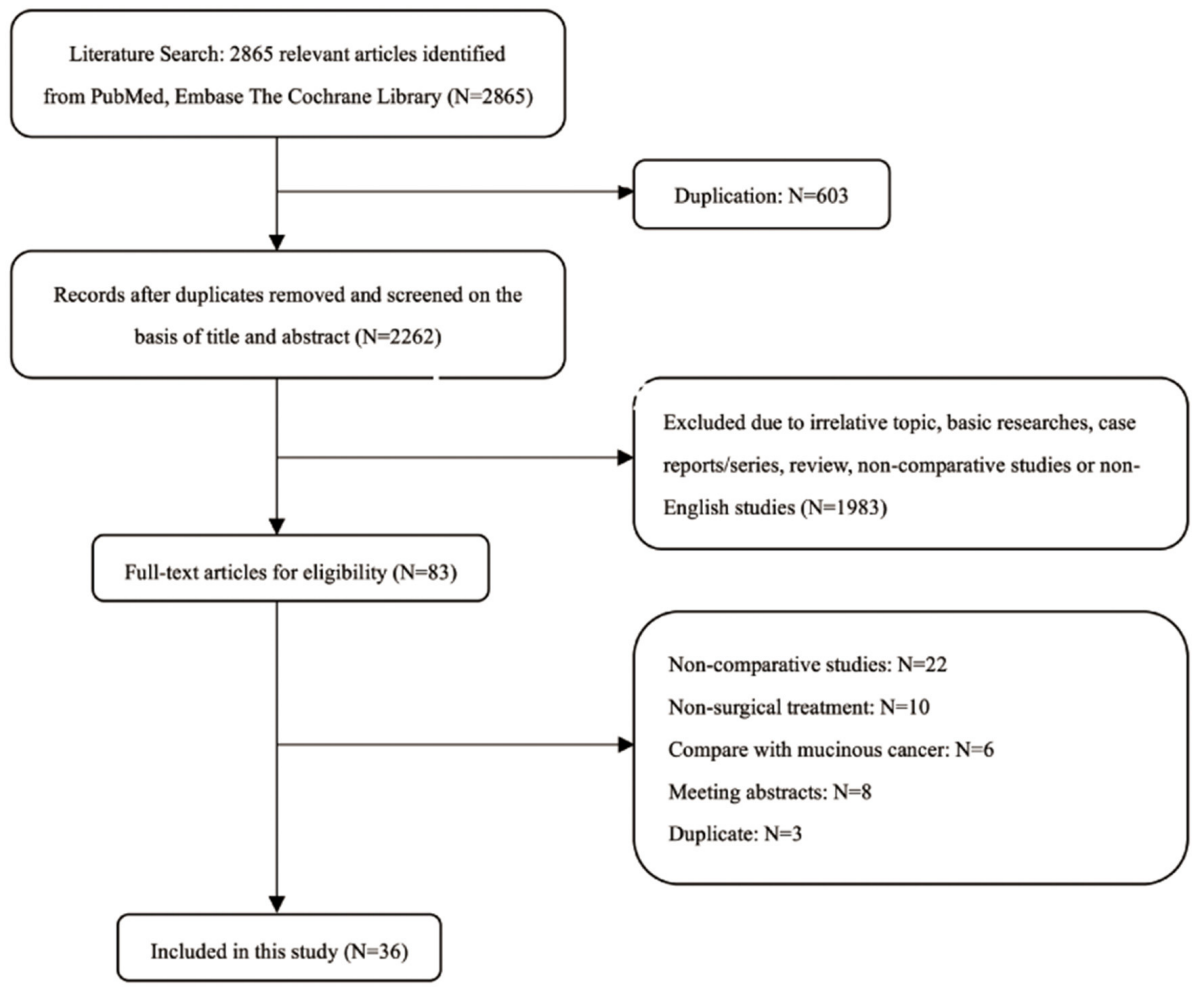
Flow chart of literature selection.

According to the selection criteria, a total of 36 studies (9,20, 24,47) with 75,116 patients (19,174 patients in the SRCC group and 55,942 patients in the NSRCC group) were included in the final meta-analysis (Figure 1). The general characteristics of those 36 studies included are presented in Table 1. These studies were from six countries and published from 1992 to 2020 and include gastric cancer patients underwent surgical treatment from 1965 to 2015. Only 9 studies included early gastric cancer (EGC) patients (12, 16, 29, 33, 38, 39, 43, 44, 46), 2 studies included only advanced gastric cancer (AGC) patients (19, 35), 18 studies included Stage IIV patients (9, 10, 13,15, 17, 18, 24, 26,28, 30,32, 34, 41, 42, 47), and 7 studies included stage IIII gastric cancer patients (11, 20, 25, 36, 37, 40, 45). The majority of these studies adopted the WHO histological classification of gastric cancer in the diagnosis of SRCC (5, 6), whereas only one study (17) used the Japanese classification (48). For the comparative group, 10 studies grouped the NSRCC gastric cancer patients according to the tumor-differentiated degree (9, 11, 12, 24, 27, 31, 35, 39, 44, 45), and the other 26 studies did not specify the composition-differentiated degree of NSRCC group. Besides, there were only one study presented that mucinous cancer was also included in the NSRCC group (27).

**Table 1:** Characteristics of the included studies.

**References**	**Period**	**Country**	**No. of Patients**	**SRCC *N* = (%)**	**NSRCC *N* = (%)**	**Stage**	**Pathological**	**Comparative group**	**NOS**
Maehara et al. (14)	19651985	Japan	1,500	51 (3.4)	1,449 (96.6)	IIV	WHO	NSRCC	8
Kim et al. (24)	19811991	Korea	3,702	450 (12.1)	3,252 (87.9)	IIV	WHO	WD, MD, PD	8
Otsuji et al. (15)	19701994	Japan	1,498	154 (10.3)	1,344 (89.7)	IIV	WHO	NSRCC	7
Yokota et al. (17)	19851995	Japan	683	93 (13.6)	590 (86.4)	IIV	Japanese^*^	NSRCC	5
Theuer et al. (25)	19841994	USA	3,020	464 (15.3)	2,556 (84.7)	IIII	WHO	NSRCC	5
Kim et al. (18)	19821999	Korea	2,358	204 (8.7)	2,154 (91.3)	IIV	WHO	NSRCC	8
Kunisaki et al. (26)	19801998	Japan	1,113	174 (15.6)	939 (84.4)	IIV	WHO	NSRCC	8
Li et al. (19)	19872003	Korea	4,759	662 (13.9)	4,097 (86.1)	AGC	WHO	NSRCC	9
Park et al. (27)	19832002	Korea	2,275	251 (11)	2,024 (89)	IIV	WHO	WMD, PD, MC	9
Piessen et al. (28)	19962007	Fance	159	59 (37.1)	100 (62.9)	IIV	WHO	NSRCC	9
Lee et al. (29)	20012008	Korea	1,362	448 (32.8)	914 (67.2)	EGC	WHO	NSRCC	7
Zhang et al. (30)	19932003	China	1,439	218 (15.1)	1,221 (84.9)	IIV	WHO	NSRCC	8
Zheng et al. (31)	19932006	China	511	39 (7.6)	472 (92.4)	IIV	WHO	WD, MD, PD	6
Chiu et al. (32)	19942006	China	2,439	505 (20.7)	1,934 (79.3)	IIV	WHO	NSRCC	9
Jiang et al. (13)	19802004	China	2,315	211 (9.1)	2,104 (90.9)	IIV	WHO	NSRCC	9
Taghavi et al. (10)	20042007	USA	10,246	2,666 (26)	7,580 (74)	IIV	WHO	NSRCC	8
Gronnier et al. (16)	19972010	Fance	421	104 (24.7)	317 (75.3)	EGC	WHO	NSRCC	7
Huh et al. (33)	19992005	Korea	2,052	540 (26.3)	1,512 (73.7)	EGC	WHO	NSRCC	7
Nafteux et al. (34)	19902009	Belgium	920	114 (12.3)	806 (87.7)	IIV	WHO	NSRCC	8
Shim et al. (20)	19982005	Korea	2,643	377 (14.2)	2,266 (85.8)	IIII	WHO	NSRCC	9
Bombat et al. (11)	19902009	USA	569	210 (36.9)	359 (63.1)	IIII	WHO	WMD, PD	8
Kim et al. (12)	19892000	Korea	2,050	345 (16.8)	1,705 (83.2)	EGC	WHO	WD, MD, PD	7
Kwon et al. (9)	19992009	Korea	769	108 (14)	661 (86)	IIV	WHO	WMD, PD	9
Zu et al. (35)	19972007	China	741	44 (5.9)	697 (94.1)	AGC	WHO	WD, MD, PD	7
Liu et al. (36)	20002008	China	1,464	138 (9.4)	1,326 (90.6)	IIII	WHO	NSRCC	9
Postlewait et al. (37)	20002012	USA	768	312 (40.6)	456 (59.4)	IIII	WHO	NSRCC	9
Wang et al. (38)	19942008	China	334	115 (34.4)	219 (65.6)	EGC	WHO	NSRCC	7
Guo et al. (39)	20022013	China	1,067	198 (18.5)	869 (81.5)	EGC	WHO	WMD, PD	7
Kong et al. (40)	19962012	China	480	90 (18.7)	390 (81.3)	IIII	WHO	NSRCC	7
Lu et al. (41)	19942013	China	2,199	354 (16.1)	1,845 (83.9)	IIV	WHO	NSRCC	7
Voron et al. (42)	19972010	Fance	1,799	899 (49.9)	900 (50.1)	IIV	WHO	NSRCC	9
Imamura et al. (43)	20062012	Japan	746	190 (25.4)	556 (74.6)	EGC	WHO	NSRCC	7
Lai et al. (44)	19872005	China	2,873	745 (25.9)	2,128 (74.1)	EGC	WHO	WD, MD, PD	6
Chon et al. (45)	20012010	Korea	7,667	1,646 (21.4)	6,021 (78.6)	IIII	WHO	WMD, PD	9
Chen et al. (46)	20022015	China	112	28 (25.0)	84 (75.0)	EGC	WHO	NSRCC	6
Chu et al. (47)	20042015	China	6,063	5,968 (98.4)	95 (1.6)	IIV	WHO	NSRCC	9

*SRCC, signet ring cell carcinoma; NSRCC, non-signet ring cancer cell; AGC, advanced gastric cancer; EGC, early gastric cancer; WD, well differentiated; MD, moderately differentiated; PD, poorly differentiated; WMD, well-moderately differentiated*.

*WHO, Histologic type of stomach cancer by WHO classification (5, 6)*.

* *Japanese, The general rules for the gastric cancer study in surgery and pathology. Part I. Clinical classification (48)*.

### Clinicopathological Characteristics

We performed pooled analysis to compare the clinicopathological characteristics between the SRCC and NSRCC patients (Table 2). Finally, we found that SRCC patients have younger age (MD: 4.90, 95% CI 5.99 to 3.82; *P* < 0.001), fewer male patients (OR: 0.55, 95% CI: 0.500.61, *P* < 0.001), less upper1 third lesions (OR: 0.62, 95% CI: 0.500.76, *P* < 0.001), more Borrmann-type IV tumors (OR: 2.47, 95% CI: 1.324.64, *P* = 0.005), and patients with distal metastasis (OR: 1.17, 95% CI: 1.081.26, *P* < 0.001) with the comparison with NSRCC patients. There was no significant difference between SRCC and NSRCC patients with regard to radical surgical resection (R0) rate (*P* = 0.25), tumor size (*P* = 0.87), proportion of advanced gastric cancers (*P* = 0.12), serosa invasive tumors (*P* = 0.71) and with lymph nodes metastasis (*P* = 0.07).

**Table 2:** The meta-analysis of clinicopathological characteristics between SRCC and NSRCC patients.

**Characteristics**	**No. of study**	**No. of SRCC**	**No. of NSRCC**	**Test of heterogeneity**		**Model**	**Meta-analysis**		
				**I^**2**^ (%)**	***P*-value**		**OR or MD**	**(95% CI)**	***P*-value**
Age (years)	17	10,590	32,739	95	<0.001	Random	4.90^*^	5.96, 3.82	<0.001
Sex (male)	36	16,386	56,013	82	<0.001	Random	0.55	0.50, 0.61	<0.001
Locations (upper)	25	10,902	48,408	89	<0.001	Random	0.62	0.50, 0.76	<0.001
Borrmann type (type-IV)	9	2,447	11,416	92	<0.001	Random	2.47	1.32, 4.64	0.005
R_0_ resection	11	3,182	14,903	90	<0.001	Random	0.81	0.56, 1.16	0.25
Tumor size (cm)	17	8,915	28,036	97	<0.001	Random	0.03^*^	0.36, 0.30	0.87
Advanced stage (T2T4 stage)	17	7,602	30,718	97	<0.001	Random	0.74	0.51, 1.08	0.12
Serosa invasive (T4 stage)	19	8,527	35,167	87	<0.001	Random	1.04	0.84, 1.28	0.71
Lymph nodes metastasis (N+ stage)	29	14,352	44,271	94	<0.001	Random	0.82	0.62, 1.02	0.07
Distal metastasis (M1 stage)	8	6,543	14,222	18	0.29	Random	1.17	1.08, 1.26	<0.001

*SRCC, signet ring cell carcinoma; NSRCC, non-signet ring cancer cell; OR, odds ratio; MD, mean difference*.

* *Mean difference (MD) was used to evaluated*.

Due to consideration that tumor stage may have interaction with the clinicopathological characteristics, subgroup analyses were performed based on the clinicopathological characteristics of early gastric cancer (EGC) and advanced gastric cancer (AGC) (Table 3). The results of meta-analysis were that SRCC patients were of significantly younger age (EGC, MD: 7.95, 95% CI: 9.68 to 6.16, *P* < 0.001; AGC, MD: 3.89, 95% CI: 5.99 to 1.76, *P* < 0.001), fewer male patients (EGC, OR: 0.57, 95% CI: 0.430.75, *P* < 0.001; AGC, OR: 0.57, 95% CI: 0.440.74, *P* < 0.001), fewer upper third tumors (EGC, OR: 0.57, 95% CI: 0.410.79, *P* = 0.007; AGC, OR: 0.75, 95% CI: 0.640.87, *P* < 0.001) than NSRCC patients in both early and advanced gastric cancers. However, with regard to tumor size, there is no significant difference between SRCC and NSRCC patients in both EGC and AGC groups (*P* = 0.83 and *P* = 0.32, respectively). We also found that there was no significant difference in lymph node metastasis between SRCC and NSRCC in advanced-stage patients (OR: 0.86, 95% CI: 0.671.10, *P* = 0.23), but SRCC patients had significantly fewer lymph nodes in metastasis than NSRCC patients with early tumor stage (OR: 0.73; 95% CI: 0.560.98, *P* = 0.02). Moreover, there is no difference in the ratio of serosa invasion (OR: 1.22, 95% CI: 0.991.49, *P* = 0.06) and distal metastasis (OR: 1.08; 95% CI: 0.911.27, *P* = 0.37) between SRCC and NSRCC of advanced stage patients.

**Table 3:** The meta-analysis of clinicopathological characteristics between SRCC and NSRCC patients based on tumor stage (EGC and AGC).

**Characteristics**	**No. of study**	**No. of SRCC**	**No. of NSRCC**	**Test of heterogeneity**		**Model**	**Meta-analysis**		
				***I*^**2**^ (%)**	***P*-value**		**OR or MD**	**95% CI**	***P*-value**
**Age (years)**									
EGC	9	1,588	4,879	85	<0.001	Random	7.95^*^	9.68, 6.16	<0.001
AGC	7	1,419	11,202	84	<0.001	Random	3.89^*^	5.99, 1.76	<0.001
**Sex (male)**									
EGC	16	3,460	11,411	90	<0.001	Random	0.57	0.43, 0.75	<0.001
AGC	9	1,744	14,440	82	<0.001	Random	0.57	0.44, 0.74	<0.001
**Tumor location (upper)**									
EGC	10	2,908	10,180	64	0.0006	Random	0.57	0.41. 0.79	0.007
AGC	14	1,788	15,137	9	0.36	Fixed	0.75	0.64, 0.87	<0.001
**R**_**0**_ **resection**									
AGC	4	802	6,446	60	0.06	Random	0.80	0.65, 0.99	0.04
**Tumor size (cm)**									
EGC	7	1,433	4,287	71	0.002	Random	0.02^*^	0.25, 0.20	0.83
AGC	6	1,362	10,816	58	0.04	Random	0.17^*^	0.16, 0.50	0.32
**Serosa invasive (T4 stage)**									
AGC	17	5,507	22,323	81	<0.001	Random	1.22	0.99, 1.49	0.06
**Lymph nodes metastasis (N+** **stage)**									
EGC	13	2,368	7,984	54	0.01	Random	0.73	0.56, 0.95	0.02
AGC	10	1,788	15,137	74	<0.001	Random	0.86	0.67, 1.10	0.23
**Distal metastasis (M1 stage)**									
AGC	5	933	7,737	57	0.05	Random	1.08	0.91, 1.27	0.37

*SRCC, signet ring cell carcinoma; NSRCC, non-signet ring cancer cell; AGC, advanced gastric cancer; EGC, early gastric cancer; OR, odds ratio; MD, mean difference*.

* *Mean difference (MD) was used to evaluated*.

### Survival Outcomes

**Figure 2:**
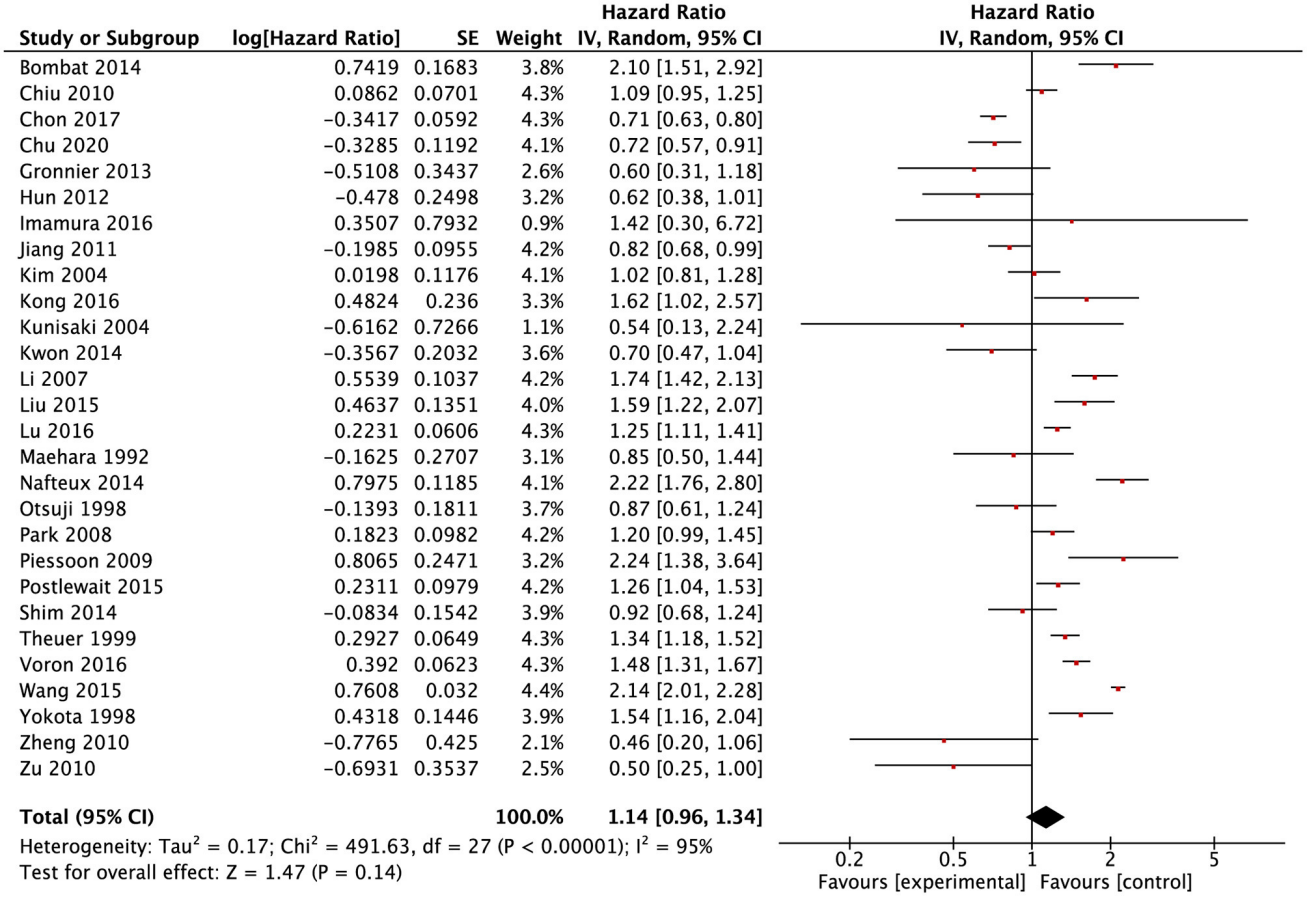
Forest plots showing the results of meta-analysis compared between SRCC patients and NSRCC patients.

**Figure 3:**
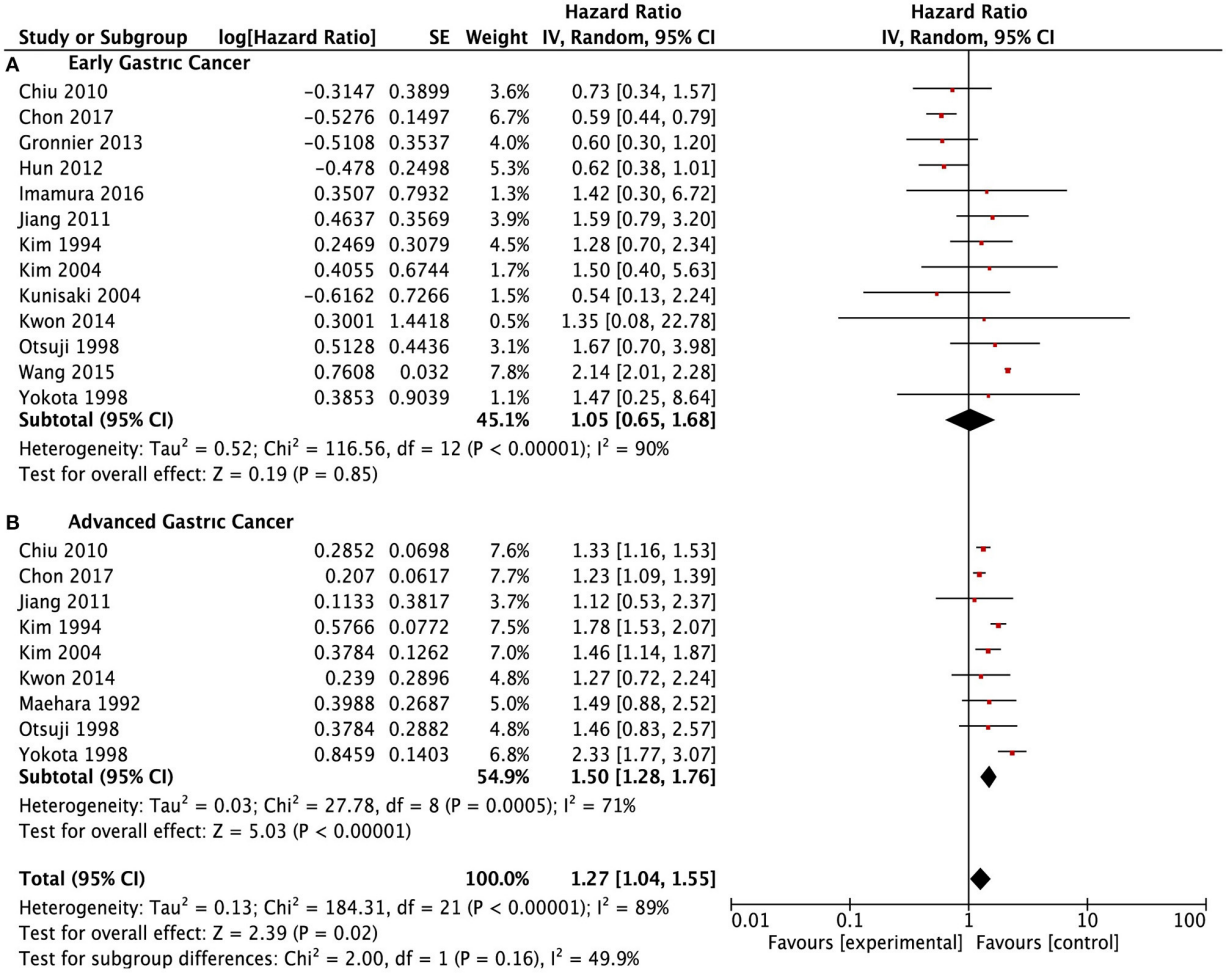
Forest plots showing the survival outcomes of meta-analysis comparing the early gastric cancers and advanced gastric cancers between SRCC and NSRCC patients. **(A)** Early gastric cancer. **(B)** Advanced gastric cancer.

A total of 28 studies (9, 11, 13,20, 24,28, 30,34, 36,38, 40,42, 45, 47) reported data of survival outcomes and included prognostic meta-analysis (Figure 2). In the pooled analysis, we found that there was a positive survival difference in SRCC patients compared with NSRCC patients (HR: 1.14, 95% CI: 0.961.34, *P* < 0.001) and with significant heterogeneity (*I*^2^ = 95%, *P* < 0.001). In view of the effect of the tumor stage on prognosis and different stage composition of different studies, subgroup survival analysis based on the different tumor stages was performed. For early gastric cancer patients, the meta-analysis included results of 13 studies (9, 13,18, 24, 26, 32, 33, 38, 45), and the results have shown that SRCC patients had similar survival outcomes with the NSRCC patients (HR: 1.05, 95% CI: 0.651.68, *P* < 0.001) (Figure 3A). For the pooled analysis of advanced gastric cancer patients (9, 13,15, 17, 18, 24, 32, 45), SRCC patients had significantly more negative survival outcomes than NSRCC patients (HR: 1.50, 95% CI: 1.281.76, *P* < 0.001) (*I*^2^ = 71%, *P* < 0.001) (Figure 3B).

**Figure 4:**
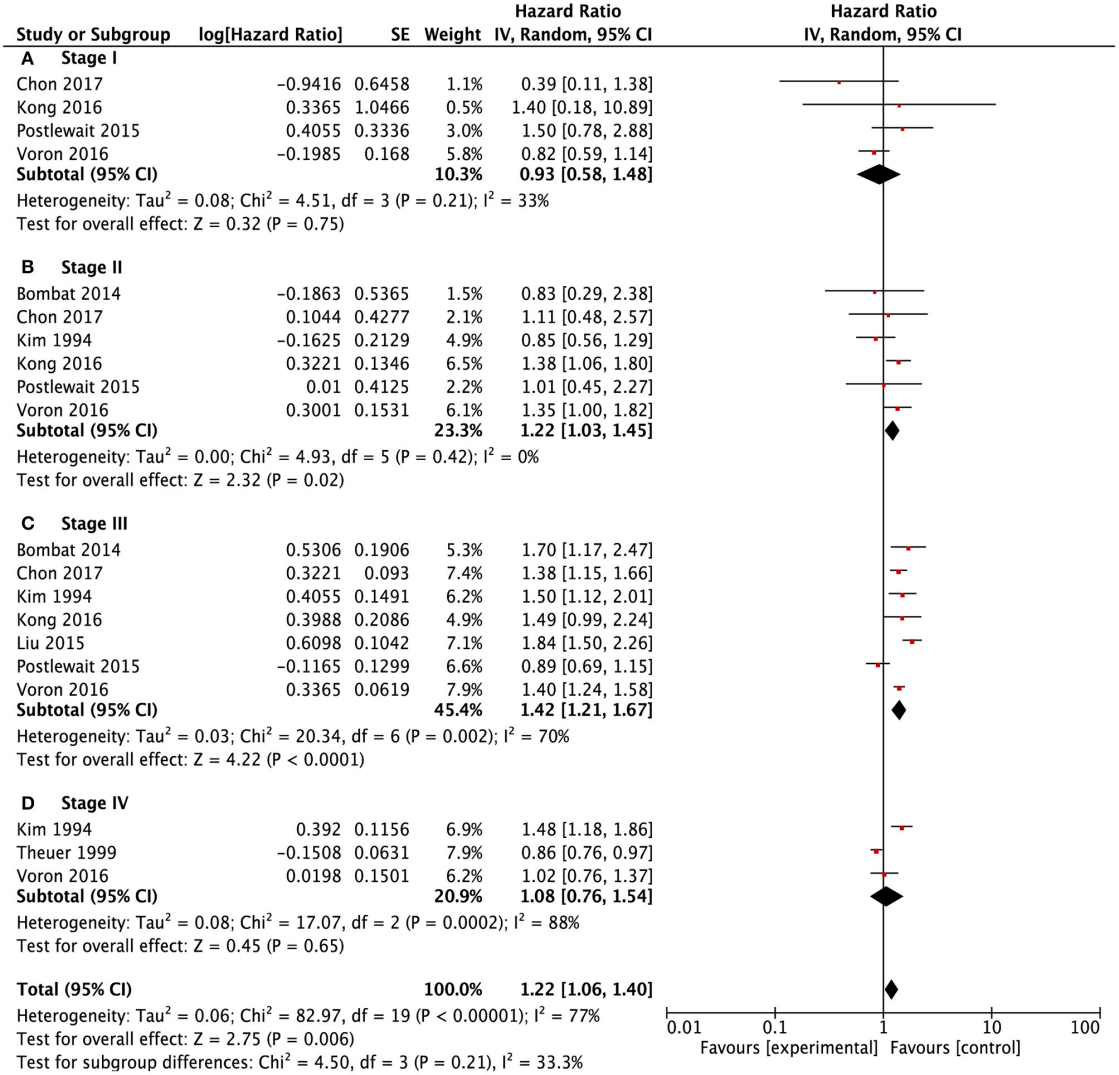
Forest plots showing the survival outcomes of meta-analysis comparing different TNM stages been SRCC and NSRCC patients. **(A)** TNM Stage I. **(B)** TNM Stage II. **(C)** TNM Stage III. **(D)** TNM Stage IV.

Meanwhile, we conducted subgroup survival analysis according to the TNM stage systems (Figure 4). SRCC and NSRCC had no significant difference in survival outcomes for stage I patients (HR: 0.93, 95% CI: 0.581.48, *P* = 0.75) and stage IV patients (HR: 1.08, 95% CI: 0.761.54, *P* = 0.21). There were significantly poorer survival outcomes of SRCC patients than NSRCC patients with TNM stage II (HR: 1.22, 95% CI: 1.031.45, *P* = 0.02) and TNM stage III (HR: 1.42, 95% CI: 1.211.67, *P* < 0.001).

### Publication Bias

Meta-regression was performed to illuminate the origin of heterogeneity. We examined the year of publication, sample size, region of study, and tumor stage in a meta-regression model. The resulting analyses indicated that publication year (*P* = 0.039) and stage of the tumor (*P* = 0.002) were significant sources of heterogeneity for overall survival outcomes (Table 4).

**Table 4:** Meta-regression for all included studies.

**Characteristics**		**Univariate analysis**	**Multivariate analysis**
		***P*-value**	***P*-value**
Publication year	19922020	0.043	0.039
Sample size	<1,000, 1,000 but <3,000, 3,000	0.407	
Region	China, Korea and Japan, Europe and North America	0.042	0.427
Tumor stage	EGC and other	0.008	0.002

*OR, odds ratio; CI, Confidence interval; EGC, early gastric cancer*.

**Figure 5:**
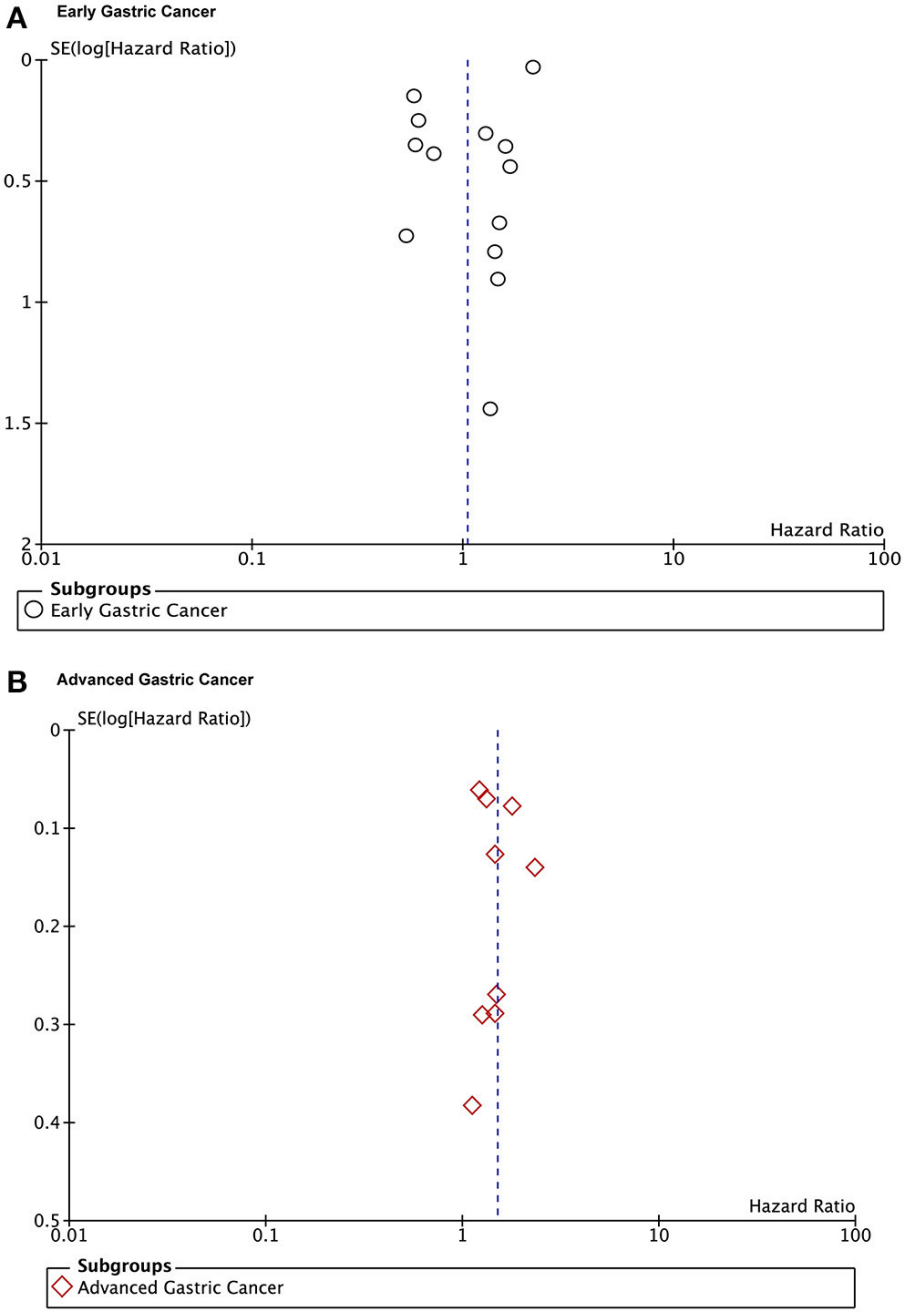
Funnel plots of the overall survival outcomes. **(A)** Early gastric cancers. **(B)** Advanced gastric cancers.

**Figure 6:**
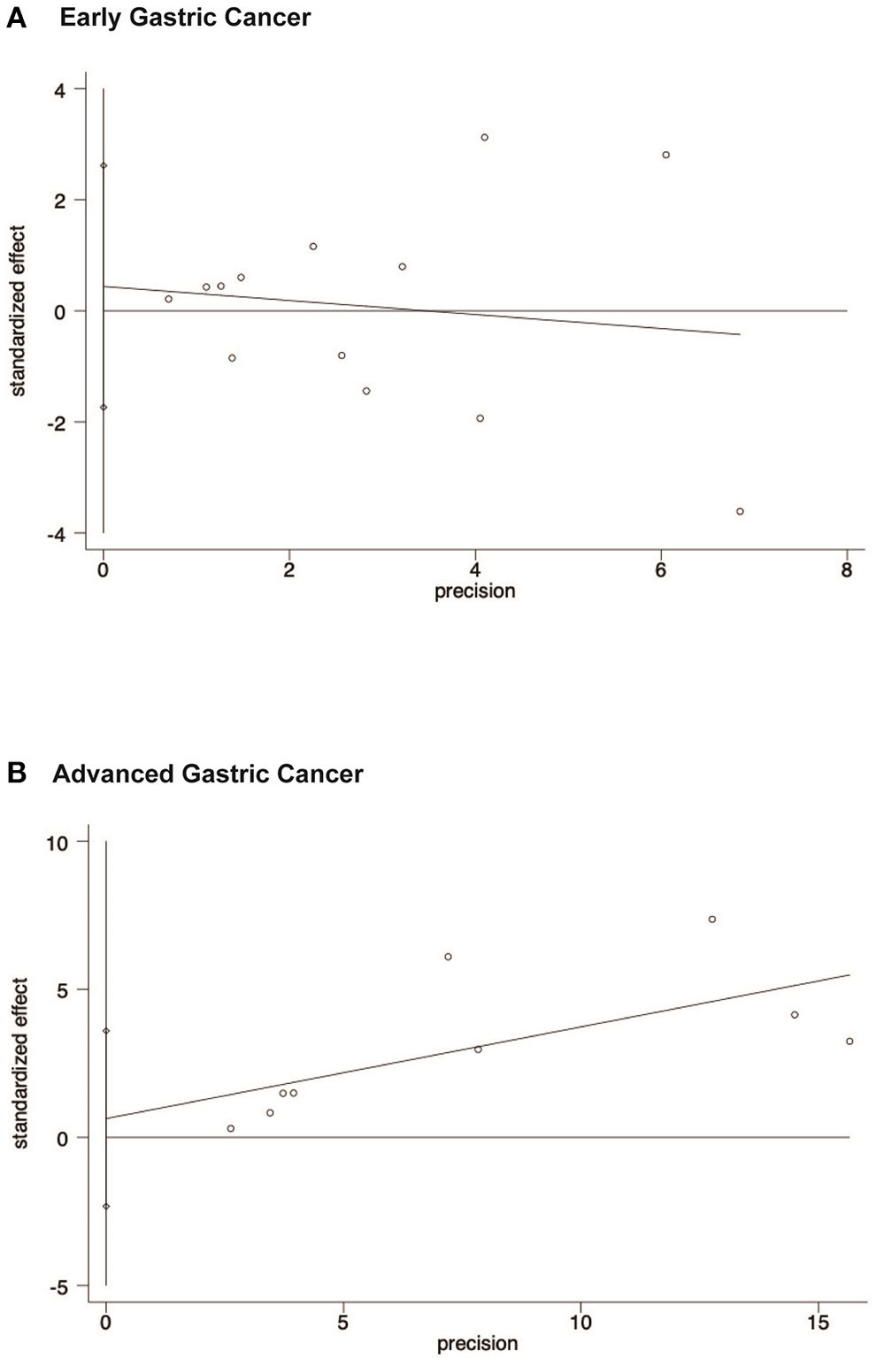
Egger's test results showing that there is no publication bias of early gastric cancer subgroup and advanced gastric cancer subgroup for overall survival outcomes. **(A)** Early gastric cancers. **(B)** Advanced gastric cancers.

The publication bias is evaluated by Funnel plots and Egger's test. The result found there was no publication bias for the early gastric cancer subgroup (*P* = 0.667) or the advanced gastric cancer subgroup (*P* = 0.629) for overall survival outcomes. The funnel plot and results of Egger's test of the early gastric cancer and advanced gastric cancer subgroup are presented in Figures 5A,B and Figures 6A,B.

## Discussion

Gastric cancer is one of the most common malignant diseases of the digestive system all over the world, and East Asian countries, such as Japan, Korea, and China have the highest incidence (1,4). Although the incidence of gastric cancer is declining, an increasing trend of signet ring cells in gastric cancer was obvious in recent decades (49). According to the previous report, signet ring cell carcinoma of the stomach has significantly different clinicopathological characteristics from other histological subtypes of gastric cancer (11,13). However, the prognostic meaning of SRCC is still controversial; for example, SRCC is a poor prognostic risk factor for overall survival outcomes (9,11). In the results of the present study, SRCC patients tended to be younger, more proportionally female, and more afflicted with middle and lower third tumors than NSRCC. As for survival outcomes, there were entirely different long-term survival outcomes of different tumor stages of SRCC when compared with NSRCC.

It is an acknowledgment that the SRCC patients are lower in age than NSRCC patients, and only a few studies reported that the mean age was similar between SRCC and NSRCC patients (19, 30, 40). In the pooled analysis, age was significantly lower for SRCC patients than NSRCC patients. Besides, we also found early-stage cancer patients have greater age variance than advanced stage patients between the SRCC and NSRCC groups. Younger cohorts, tend to have a greater proportion of female patients, which is another clinicopathological characteristic of SRCC patients. However, the essential reason for a high proportion of female patients is unclear. Some studies have concluded that this phenomenon is due to the sex hormones of SRCC patients (50, 51).

We conducted an analysis of lymph node metastasis of SRCC and NSRCC patients. The results were that there was no significant difference for advanced gastric cancer patients between SRCC and NSRCC patients (OR: 0.86, 95% CI: 0.671.10, *P* = 0.23). However, for early gastric cancer patients, the results showed that SRCC patients had significantly lower incidence of lymph node metastasis than NSRCC patients. These results are consistent with results of the previous clinical study, in which Korean scholars deemed that the lymph node metastasis risk is low when the SRCC tumor was confined in the mucosa layer, but the risk of lymph node metastasis increases significantly, once the tumor penetrates the submucosa layer to the deep layers (52, 53).

The dispute about survival outcomes of SRCC patients is a major controversy when compared with NSRCC patients. During recent decades, scholars generally consider that SRCC patients have poorer survival outcomes than NSRCC patients, due to poor tumor behavior. However, the studies published in recent years have reported that the survival outcomes of SRCC patients should be evaluated and adjusted by tumor stage (20). For early gastric cancer, majority studies reported that SRCC was a good prognostic factor (15, 24, 43, 45). Besides, some also reported that the survival outcomes were comparable between SRCC and NSRCC patients (13, 17). In the pooled analysis of our study, SRCC patients have similar survival outcomes to those of NSRCC patients in both early gastric cancer patients and stage-I patients and with low heterogeneity. At least we can show that, for early gastric cancers, the long-term prognosis of SRCC patients is not worse than that of NSRCC patients. It needs to be mentioned that the present study only included patients who underwent surgical treatment. Those SRCC and NSRCC patients who had endoscopic mucosa resection or endoscopic submucosa dissection are not included in this study.

For advanced gastric cancer patients, the prognostic meaning of signet ring cancer cell content is controversial. The general consensus is that the SRCC patients had poorer survival outcomes than the NSRCC patients (19). But does the evidence support this consensus? Some scholars claimed that SRCC patients had similar survival outcomes as NSRCC patients, and the survival evaluation between SRCC and NSRCC patients should adjust the differentiated degree and tumor stage (11, 45). A Korean study found that SRCC and NSRCC patients had similar survival outcomes after adjusting for the tumor stage by propensity score matching (20). In the pooled survival outcomes of advanced tumor stage patients, we found SRCC patients had significantly poorer survival outcomes than NSRCC patients (HR 1.27, 95% 1.041.55). However, according to the TNM staging system of gastric cancer, advanced gastric cancers included tumors with T24, N/+, Mx stages. Therefore, we performed a survival analysis according to the TNM stage, and we found that SRCC patients had similar survival outcomes in stage I and stage IV patients, and poorer survival outcomes in stage II and stage III SRCC patients with the comparison with NSRCC patients. Therefore, the prognosis of stage I and stage IV SRCC patients can be considered almost equal to that of NSRCC patients; but for the locally advanced stage (stages IIIII) patients, the prognosis of SRCC patients is significantly poorer than for NSRCC patients.

Most of the studies included were retrospective studies. The quality of different retrospective studies varies, which is inevitable. Because of this, we use the NOS scoring system to evaluate the quality of each study included. Among the 36 retrospective studies included, two had a NOS score of 5. We eliminated these two studies with relatively poor quality and conducted a subgroup analysis. In the end, we found that the results were not statistically different from those before the elimination. Through careful statistical analysis, 36 studies were finally included.

There is no consistent evidence about the appropriate chemotherapy treatment strategies for signet ring cell gastric carcinoma to improve prognosis. In previous studies, signet ring cell gastric carcinoma of the stomach was generally considered to be insensitive to chemotherapy, but there was no definite clinical evidence to support it. The comparison of chemosensitivity between signet ring cell gastric carcinoma and non-signet ring cell gastric carcinoma is still limited. Our previous study found that not all signet ring cell gastric cancers were insensitive to chemotherapy, and its chemosensitivity was related to the CLDN18-ARHGAP26/6 fusion gene (54). Li explored the survival of stage IIIII primary signet ring cell gastric carcinoma by adjuvant chemoradiotherapy (55). In this study, SRCC patients with stage IIIII experienced improved overall survival after receiving adjuvant chemoradiotherapy, which provides several treatment implications. Therefore, more clinical trials will be needed to verify the conclusion.

However, there were several limitations in the present meta-analysis. First, all studies included are associated with long time spans and different versions of tumor stage classification. The stage migration and corresponding outcomes bias were exactly included among these studies and may result in the high heterogeneity in the pooled analysis. Second, the studies included were from different countries, the different treatment strategies from eastern and western countries were bias factors. Besides, different stage compositions between eastern and western countries also have an influence on the survival analysis. Third, all of the studies included are retrospective studies. The natural limitation and quality of the retrospective studies were another factor resulting in bias. Fourth, there is no indication of radical surgery for stage IV gastric cancer. And the reason for surgery is mostly because of complications caused by tumors such as bleeding and obstruction, rather than the tumor itself. So fewer patients with stage IV gastric cancer were included. The heterogeneity test has been completed, and its purpose is to minimize the impact that heterogeneity may have on the quality of research and results.

## Conclusions

Signet ring cell carcinoma of the stomach is one of the specific histological types of gastric carcinomas. The signet ring cell gastric cancer is predominantly found among younger people and females than non-signet ring cell gastric cancer. The prognostic features of signet ring cell carcinoma are significantly correlated with tumor stage. For gastric cancer patients with T1 stage or TNM stage-I, the prognosis of SRCC patients is comparable to that of NSRCC patients. For patients with T2T4 stages and TNM stages IIIII, the prognosis of SRCC patients is significantly worse than for NSRCC patients.

## Data Availability Statement

The original contributions presented in the study are included in the article/supplementary material, further inquiries can be directed to the corresponding author/s.

## Author Contributions

W-HZ and J-KH designed the study. CZ, RL, W-HZ, X-ZC, KL, KY, X-LC, L-YZ, Z-XC, Z-GZ, and J-KH collected information and analyzed and interpreted the data. W-HZ and J-KH supervised this study. All authors contributed to the article and approved the submitted version.

## Conflict of Interest

The authors declare that the research was conducted in the absence of any commercial or financial relationships that could be construed as a potential conflict of interest.
